# Evaluation of Dried Plasma Spot‐Based Quantification of Glial Fibrillary Acidic Protein as a Disease‐Associated Biomarker in Neuromyelitis Optica Spectrum Disorder

**DOI:** 10.1002/acn3.70504

**Published:** 2026-07-28

**Authors:** Felix Wohlrab, Evelyn Alvarez, Roua Hamdi, Bingqing Zhang, Hayeun Ji, Niyati Jhaveri, Patrick Schindler, Pedro Sanchez, Pia S. Sperber, Tanja Schmitz‐Hübsch, Frederike C. Oertel, Nisa Vorasoot, Najib Kissani, Falko Böhringer, Jens Kuhle, Anne‐Katrin Pröbstel, Xiao‐Jun Ma, Péter Körtvelyessy, Patrick Waters, Friedemann Paul

**Affiliations:** ^1^ Experimental and Clinical Research Center (ECRC) Charité‐Universitätsmedizin Berlin, Corporate Member of Freie Universität Berlin, Humboldt‐Universität zu Berlin Berlin Germany; ^2^ Alamar Biosciences Fremont, California USA; ^3^ Neuroscience Clinical Research Center (NCRC) Charité‐Universitätsmedizin Berlin, Corporate Member of Freie Universität Berlin, Humboldt‐Universität zu Berlin Berlin Germany; ^4^ Department of Neurology Charité‐Universitätsmedizin Berlin, Corporate Member of Freie Universität Berlin and Humboldt‐Universität zu Berlin Berlin Germany; ^5^ Einstein Center Digital Future Berlin Berlin Germany; ^6^ Division of Neurology, Department of Medicine, Faculty of Medicine Khon Kaen University Khon Kaen Thailand; ^7^ Laboratory of Clinical and Experimental Neuroscience, Faculty of Medicine Cadi Ayyad University Marrakech Morocco; ^8^ Labor Berlin – Charité Vivantes Services GmbH Berlin Germany; ^9^ Multiple Sclerosis Center and Research Centers for Clinical Neuroimmunology and Neuroscience (RC2NB) Neurology University and University Hospital Basel Basel Switzerland; ^10^ Department of Biomedicine and Clinical Research University Hospital and University of Basel Basel Switzerland; ^11^ Center for Neurology, Department of Neuroimmunology and Neuromuscular Diseases University Hospital and University Bonn Bonn Germany; ^12^ German Center for Neurodegenerative Diseases (DZNE) Bonn Germany; ^13^ German Center for Neurodegenerative Diseases (DZNE) Magdeburg Germany; ^14^ Oxford Autoimmune Neurology Diagnostic Laboratory, Nuffield Department of Clinical Neurosciences University of Oxford Oxford UK

**Keywords:** clinical neuroimmunology, dried blood spot sampling, fluid biomarkers, glial fibrillary acidic protein (GFAP), Neuromyelitis optica spectrum disorder (NMOSD)

## Abstract

**Objective:**

To evaluate the diagnostic accuracy of glial fibrillary acidic protein (GFAP) measured in dried plasma spots versus conventional plasma‐ and serum‐GFAP testing for assessment of disease severity in aquaporin‐4 immunoglobulin G–positive neuromyelitis optica spectrum disorder (AQP4‐IgG^+^ NMOSD).

**Methods:**

A neuroimmunological prospective cohort of remission samples from 70 participants with the diagnoses AQP4‐IgG^+^ NMOSD (*n* = 19), myelin oligodendrocyte glycoprotein antibody–associated disease (MOGAD; *n* = 9), relapsing–remitting multiple sclerosis (RRMS; *n* = 28) and healthy controls (HC; *n* = 14) from a single center were included. GFAP concentrations were measured in frozen and thawed plasma and paired dried plasma spot (DPS) samples using an ultrasensitive proximity ligation–based assay (nucleic acid–linked immuno‐sandwich assay; NULISA). In NMOSD, GFAP was additionally quantified in paired serum samples using a single‐molecule array (Simoa) assay as a reference. Cross‐matrix correlations, diagnostic performance, and associations with neurological disability were evaluated.

**Results:**

GFAP concentrations measured by NULISA correlated strongly between plasma and DPS samples across diagnostic groups and healthy controls. In NMOSD, plasma GFAP measured by NULISA showed strong concordance with serum GFAP quantified by Simoa. DPS‐derived and plasma‐derived GFAP demonstrated good diagnostic accuracy for AQP4‐IgG^+^ NMOSD and was significantly associated with neurological disability as measured by the Expanded Disability Status Scale (EDSS). Group‐wise comparisons across plasma and DPS showed retained elevation of GFAP in AQP4‐IgG^+^ NMOSD compared with the other diagnostic groups and healthy controls.

**Interpretation:**

GFAP quantification using the NULISA platform is feasible in plasma and DPS samples and enables reliable biomarker assessment in a potentially remote‐compatible setting. DPS‐derived GFAP measurements retained meaningful information on disability in NMOSD and may provide an analytical framework for future studies evaluating minimally invasive capillary or self‐sampling approaches.

## 1 Introduction

In recent years, the development of blood‐based biomarkers for the assessment of disease activity and prognosis in neuroimmunological disorders has advanced substantially. In multiple sclerosis (MS), converging evidence has brought blood biomarkers close to routine clinical implementation. Specifically, serum neurofilament light chain (sNfL) has emerged as a robust marker of neuroaxonal injury with prognostic value [1], while glial fibrillary acidic protein (GFAP), an astrocytic intermediate filament protein, also holds promise for translation into clinical practice as a marker of chronic neuroinflammation and progression independent of relapse activity [2].

In contrast to MS, fewer studies have investigated these biomarkers in aquaporin‐4 immunoglobulin G–positive neuromyelitis optica spectrum disorder (AQP4‐IgG^+^ NMOSD), an autoantibody‐mediated astrocytopathy characterized by severe attacks and substantial disability accrual [3]. Available data consistently indicate that GFAP is of particular relevance in NMOSD, reflecting astrocytic damage and disease activity. Elevated blood GFAP levels have been shown to distinguish NMOSD from other neuroinflammatory disorders and to differentiate relapse from remission states, underscoring its diagnostic and disease‐monitoring potential [4, 5, 6, 7].

Recent work in Alzheimer's disease biomarker research has demonstrated that protein biomarkers, including GFAP, can be collected and quantified using minimally invasive blood sampling approaches such as dried blood or plasma spots [8, 9]. These developments highlight an opportunity to improve accessibility to biomarker‐based diagnostics and longitudinal disease monitoring, particularly for patients with limited mobility or restricted access to specialized care. However, despite its strong biological rationale and clinical relevance, access to GFAP testing in NMOSD in routine care remains limited, and its use in remote or home‐based settings is largely unexplored. Moreover, diagnostic gaps remain substantial in under‐resourced regions, including parts of the Global South, where NMOSD is likely underdiagnosed and access to specialized diagnostic tools is limited [10, 11, 12]. This challenge is particularly evident in parts of Africa, where NMOSD is likely substantially underdiagnosed owing to limited structural access to diagnostic infrastructure [13].

The novel ultra‐sensitive proximity ligation–based assay termed nucleic acid–linked immuno‐sandwich assay (NULISA) platform enables ultra‐sensitive protein quantification in the attomolar range and may therefore be particularly suitable for dried blood biomarker applications characterized by low analyte abundance and matrix‐related recovery effects [14].

Against this background, we investigated whether GFAP can be reliably quantified from dried plasma spot (DPS) samples using the NULISA platform. Specifically, we tested the hypotheses that (i) GFAP quantification from DPS samples is technically feasible using NULISA and evaluate its correlation to plasma levels in paired samples, and (ii) DPS‐derived GFAP levels reflect disease activity and exhibit diagnostic value for AQP4‐IgG^+^ NMOSD compared with healthy individuals and patients with other neuroimmunological diseases.

## 2 Methods

### 2.1 Clinical Cohort

**FIGURE 1:**
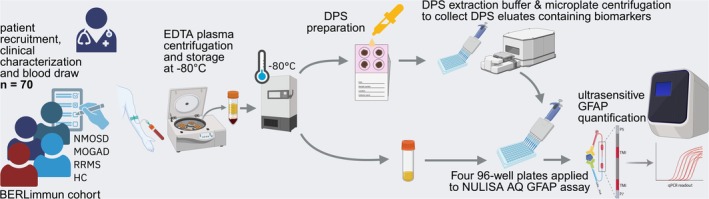
Study design and analytical workflow. Overview of participant recruitment (*n* = 70), clinical characterization, biospecimen processing and analysis. Venous blood was collected and EDTA plasma obtained by centrifugation and biobanked at –80°C. Thawed plasma was either analyzed directly or applied to filter cards for DPS preparation. DPS samples were air‐dried, subsequently extracted, and eluates collected by microplate centrifugation. GFAP concentrations in plasma and DPS eluates were quantified using the NULISA GFAP AQ singleplex assay across four 96‐well plates (created with BioRender).

This was a monocentric, observational study based on a single‐center neuroimmunological prospective cohort at Charité‐Universitätsmedizin Berlin (BERLimmun; German Clinical Trial Register DRKS00026761; [15]). The analytic workflow is summarized in Figure 1. The present analysis included patients diagnosed with AQP4‐IgG^+^ NMOSD (*n* = 19), myelin oligodendrocyte glycoprotein antibody–associated disease (MOGAD; *n* = 9), and relapsing–remitting multiple sclerosis (RRMS; *n* = 28) from 29/11/2021 to 07/04/2025. In addition, we included healthy control participants (HC; *n* = 14) derived from the same study between 6/12/2021 and 10/01/2023 to match the patient cohort with respect to age and sex at the group level. Inclusion criteria for patients comprised age ≥ 18 years at study inclusion, provision of written informed consent, and clinically stable disease under immunotherapy (anti‐CD20 therapy or dimethyl fumarate for RRMS) at the time of sampling, as confirmed by neurological examination, brain and spinal magnetic resonance imaging (MRI). Patients were considered clinically stable if they had been free of clinical relapse or MRI activity for at least 6 months prior to sampling and had received stable immunotherapy for at least 6 months. NMOSD diagnoses were established according to the international consensus criteria by Wingerchuk et al. [16], RRMS was diagnosed according to the 2017 revised McDonald criteria [16, 17]. MOGAD was diagnosed in accordance with contemporary consensus recommendations, including the criteria proposed by Banwell et al. [18]. MOGAD and NMOSD were diagnosed with support from autoantibody quantification using fixed cell‐based assays [19, 20]. HC had no history of neurological or inflammatory disease. All study participants underwent a standardized clinical assessment at the time of biospecimen collection. Recorded variables included basic demographics (age, sex, and body mass index [BMI]), clinical history, autoantibody titers and time since last clinical relapse to the date of sample collection. Neurological disability was quantified using the Expanded Disability Status Scale (EDSS). The study was approved by the Ethics Committee of Charité‐Universitätsmedizin Berlin (EA1/362/20), and all participants provided written informed consent prior to inclusion.

### 2.2 Blood Collection and DPS Preparation

Venous blood was collected by peripheral venipuncture from study participants between November 2021 and April 2025. Plasma was obtained from ethylenediaminetetraacetic acid (EDTA) vacutainer tubes (Becton, Dickinson and Company, Franklin Lakes, NJ, USA), kept on ice for 30 min, and centrifuged at 3000 × g for 10 min at 4°C. Serum was collected in serum vacutainer tubes (Becton, Dickinson and Company), allowed to clot for 30 min at room temperature, and centrifuged at 2000 × g for 10 min at 4°C. Plasma and serum were aliquoted and stored at −80°C until analysis.

For DPS preparation, biobanked plasma aliquots were thawed on ice, and 50 μL of EDTA plasma was applied to dried blood analysis filter cards (Ahlstrom Biosample Collection Cards; Ahlstrom, Helsinki, Finland) using calibrated pipettes. Subsequently, the spots were air‐dried overnight at room temperature and stored at room temperature for up to 1 week prior to extraction and analysis. Following DPS preparation, the remaining plasma was re‐frozen at −80°C and subsequently shipped on dry ice for biomarker analysis. Pre‐analytical handling was guided by published stability data for dried blood spots in biobanking contexts and data evaluating room temperature stability of proteins in dried blood samples [21, 22].

### 2.3 DPS Extraction

For DPS extraction, each filter disc was placed at the bottom of an individual well of AcroPrep 24‐well filter plates with Supor EKV membrane (Cytiva, Marlborough, MA, USA) and a corresponding elution plate was positioned beneath the filter plate. Subsequently, 200 μL of in‐house NULISA sample extraction buffer (Alamar Biosciences, Fremont, CA, USA) supplemented with protease and phosphatase inhibitors was added to each well of the filter plate. The plate stack was shaken for 1 h at 1000 rpm at room temperature, followed by centrifugation at 2200 × g for 15 min at 4°C to facilitate elution. The extracted volume was then transferred to a labeled Eppendorf tube. For samples located at the edges of the filter plate that yielded lower elution volumes, an additional manual extraction step was performed by squeezing the filter disc to recover remaining liquid.

### 2.4 Biochemical Analysis

GFAP concentrations in plasma and paired DPS samples were quantified using the novel NULISA singleplex GFAP absolute quantification (AQ) assay, enabling absolute protein quantification via a calibrator curve. This assay is an extension of the NULISA proteomic platform [14]. Four assay plates were run. Each plate included an eight‐point calibration curve run in duplicate, blank wells measured in quadruplicate, 37 biological samples measured in duplicate, and absolute GFAP concentrations were determined using the respective plate‐specific calibration curves. The analytical lower limit of quantification was 0.319 pg/mL, and the calibration range spanned 0.319 to 4990 pg/mL. Assay performance was monitored using intra‐ and inter‐plate quality control metrics. Coefficients of variation for calibrators, quality controls, and biological samples met predefined acceptance criteria of < 25%, and calibrator recovery was within the acceptable range of within ±25%. All samples yielded concentrations within the calibration range and above the assay lower limit of quantification.

Serum GFAP concentrations were measured using a single‐molecule array (Simoa) immunoassay on the Quanterix platform. Following the analytical protocol previously described by Schindler et al. [23], samples were analyzed in duplicate using calibrator‐based absolute quantification and standard quality control procedures [23].

### 2.5 Statistical Analysis

Baseline demographic and clinical variables were summarized descriptively and compared across diagnostic groups. Continuous variables were assessed using Welch's one‐way ANOVA to account for unequal variances, followed by Games‐Howell post hoc tests where appropriate. For non‐normally distributed continuous variables, group comparisons were performed using the Kruskal‐Wallis test with Dunn's post hoc test and Bonferroni correction for multiple comparisons. Categorical variables were compared using Fisher's exact test.

For GFAP analyses, 24 DPS samples exhibiting predefined pre‐analytical extraction artifacts (e.g., pronounced edge effects or manual recovery) were excluded prior to statistical evaluation. Subsequently, 46 DPS samples (*n* = 12 for NMOSD, *n* = 7 for MOGAD, *n* = 17 for RRMS and *n* = 10 for HC) entered all downstream analyses. GFAP concentrations across diagnostic groups were compared using the nonparametric Kruskal–Wallis test followed by Dunn's post hoc test with Bonferroni correction for multiple comparisons (α = 0.05), as data were non‐normally distributed and sample sizes were small. Paired comparisons between matched plasma and DPS GFAP measurements were performed using the Wilcoxon matched‐pairs signed‐rank test. DPS‐to‐plasma GFAP ratios were calculated for each paired sample, and median (IQR) values were reported. The relationship between DPS and plasma GFAP concentrations was further assessed using linear regression, including a model without intercept to estimate proportional scaling. Regression coefficients (β) and 95% confidence intervals (CI) were reported. Associations between GFAP concentrations across matrices and platforms were assessed using Pearson's correlation coefficient with linear regression lines for visualization. The rank‐order relationship between plasma and DPS GFAP concentrations was additionally evaluated using Spearman's rank correlation coefficient. Diagnostic performance of DPS‐derived GFAP for NMOSD was assessed using receiver operating characteristic (ROC) curve analysis, reporting area under the curve (AUC), 95% CI, and two‐sided *p* values.

The association between DPS GFAP concentrations and neurological disability was evaluated using linear regression with DPS GFAP as the dependent variable and the EDSS as the independent variable (DPS GFAP ~ EDSS). To account for potential confounding by age, multivariable linear regression models additionally including age as a covariate were performed (DPS GFAP ~ EDSS + age). Unstandardized regression coefficients (β), 95% CI, and two‐sided *p* values were reported. A standardized effect size (SES) was calculated as SES = β · SD(EDSS)/SD(DPS GFAP). Regression lines with 95% CI were visualized across the observed EDSS range.

All statistical tests were two‐sided, and *p* < 0.05 was considered statistically significant. Statistical analyses and visualizations were performed using R (version 4.4.3) and GraphPad Prism version 10 (GraphPad Software, Boston, MA, USA).

## 3 Results

### 3.1 Baseline Demographic and Clinical Characteristics

Baseline demographic and clinical characteristics are summarized in Table 1. A total of 46 participants were included for data analysis, comprising 12 NMOSD, 7 MOGAD, 17 RRMS, and 10 HC. Sex distribution did not differ among groups (Fisher's exact test, *p* = 0.22). Age differed across cohorts (Welch's ANOVA, *p* = 0.023), with NMOSD patients being older than MOGAD, RRMS, and HC. BMI was comparable across groups (Welch's ANOVA, *p* = 0.39). Among disease cohorts, time since the last clinical relapse did not differ between groups (Welch's ANOVA, *p* = 0.12). All patient groups were confirmed to have stable disease based on clinical examination, brain and spinal MRI. Disability as assessed by EDSS did not differ between disease groups (Kruskal–Wallis, *p* = 0.86). Current immunomodulatory treatments reflected disease‐specific management strategies. All NMOSD and MOGAD patients were treated with anti‐CD20 monoclonal antibodies. Among RRMS patients, 53% received dimethyl fumarate and 47% anti‐CD20 therapy. All HC were untreated.

**TABLE 1:** Clinical and demographic characteristics of the study participants. Values are given as mean (SD) unless otherwise indicated. Sex is expressed as the number of female and male participants. *p* values refer to overall group comparisons performed with Welch's one‐way ANOVA followed by Games‐Howell post hoc tests for continuous variables, Kruskal–Wallis test with Dunn's post hoc test for non‐normally distributed continuous variables and Fisher's exact test for categorical variables. No formal statistical comparison was performed for treatment categories, as therapeutic regimens reflect disease‐specific standards of care.

Characteristic	HC (*n* = 10)	NMOSD (*n* = 12)	MOGAD (*n* = 7)	RRMS (*n* = 17)	*p*
Sex, F/M (*n*)	7/3	11/1	3/4	7/10	0.22
Age, years	38.1 (15.9)	48.9 (16.2)	29.7 (4.6)	38.9 (9.7)	0.023
BMI, kg m^−2^	22.8 (3)	26.5 (3.9)	26.3 (6.9)	25.7 (6.5)	0.39
Time since last relapse, months	—	72.5 (47.7)	41.0 (36.6)	41.2 (40.3)	0.12
EDSS, median (IQR)	—	2 (1.5–5)	2 (2–3.5)	2 (1.5–3.5)	0.86
Treatment, *n* (%)	—	—	—	—	—
Anti‐CD20 therapy	—	12 (100)	7 (100)	8 (47)	—
Dimethyl fumarate	—	—	—	9 (53)	—
None	10 (100)	—	—	—	—

### 3.2 DPS Extraction

DPS extraction was technically feasible and yielded analyzable samples across all specimens, although matrix‐specific constraints were observed. The Ahlstrom filter discs used for DPS preparation exhibited high absorbency, leading to partial retention of extraction buffer within the matrix and recovery of approximately 25% of the initial input volume (~40–65 μL were eluted from 200 μL input volume). Eluates were primarily obtained by centrifugation using a filter plate. During the centrifugation process, a plate‐edge effect was observed for *n* = 24 samples (34%), with reduced extraction efficiency in peripheral wells of the filter plate. To optimize sample yield for this subset of edge‐position samples, additional manual recovery was required. All *n* = 24 (34%) samples exhibiting pronounced edge effects, requiring manual recovery were excluded from downstream data analyses. This led to sample exclusion of *n* = 7 NMOSD (33.3% of NMOSD), *n* = 2 MOGAD (20% of MOGAD), *n* = 11 RRMS (27.5% of RRMS), and *n* = 4 HC (26.7% of HC). Following extraction, duplicate GFAP measurements were performed using a 20 μL input volume per replicate.

### 3.3 Correlation of Plasma and DPS GFAP Values Over All Groups Across Matrices

**FIGURE 2:**
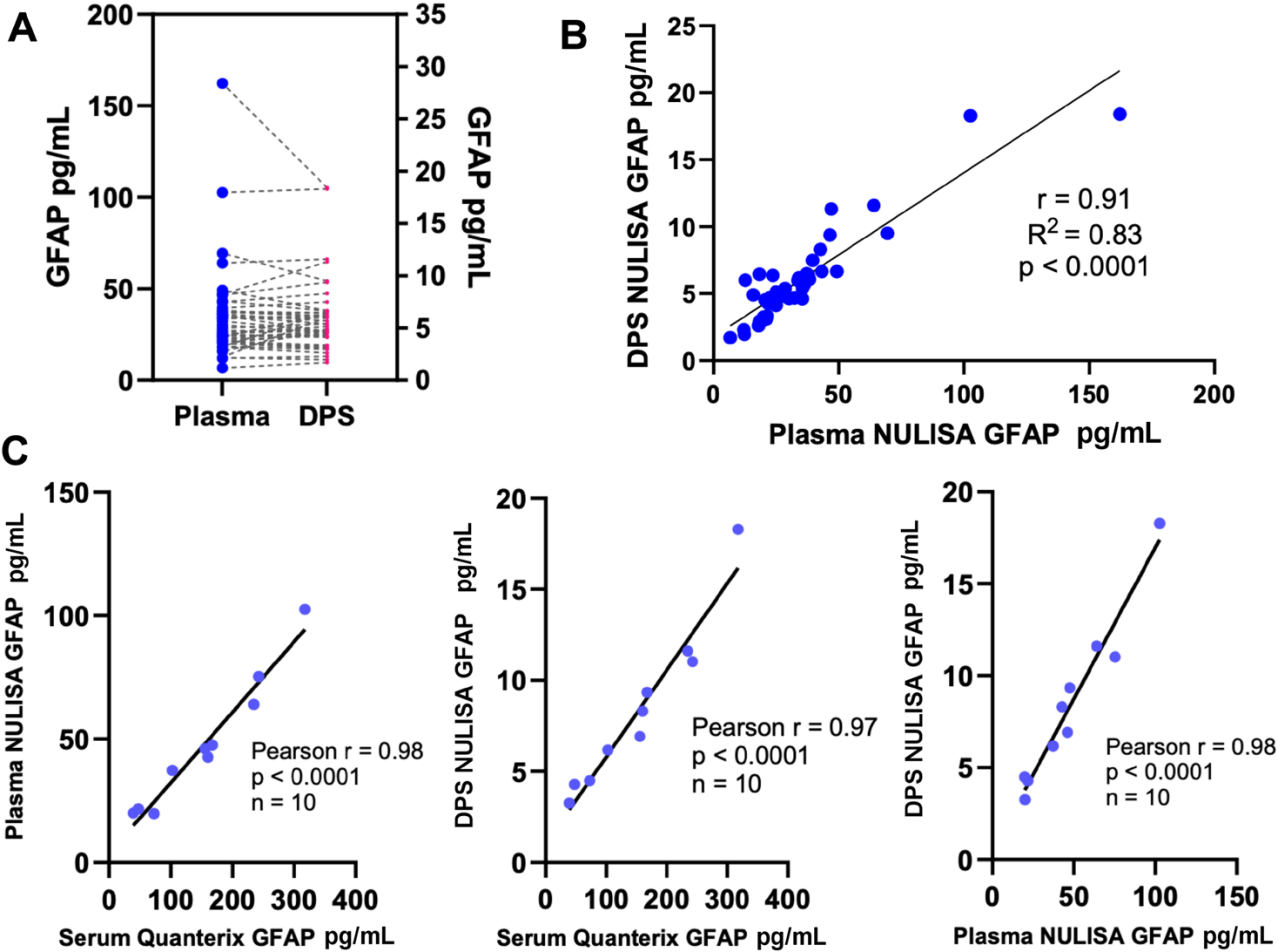
Comparability of GFAP measurements across matrices and assay platforms. (A) Paired comparison of GFAP concentrations measured in plasma and DPS samples (*n* = 46). GFAP concentrations were consistently lower in DPS than in matched plasma samples (Wilcoxon matched‐pairs signed‐rank test, *p* < 0.001; median paired difference −21.3 pg/mL), while rank‐order agreement between matrices remained high (Spearman ρ = 0.82, *p* < 0.001). (B) Correlation between mean duplicate GFAP concentrations measured in plasma and DPS across diagnostic groups (RRMS, NMOSD, MOGAD, and HC), demonstrating a strong linear association (Pearson *r* = 0.91, *R* ^2^ = 0.83, *p* < 0.001). (C) Cross‐platform correlation of GFAP concentrations in NMOSD (*n* = 10) measured by Simoa (Quanterix) in serum and NULISA in plasma and DPS samples. Lines indicate linear regression fits; Pearson correlation coefficients (r), and two‐tailed *p* values are shown.

To assess the comparability of GFAP concentrations between plasma and DPS samples, paired GFAP measurements were analyzed across all diagnostic groups. GFAP concentrations were consistently lower in DPS than in matched plasma samples across all individuals (*n* = 46), as demonstrated by a Wilcoxon matched‐pairs signed‐rank test (*p* < 0.001; median paired difference −21.3 pg/mL; Figure 2A). Despite this systematic difference in absolute concentrations, rank‐order agreement between matrices was high, with a strong Spearman correlation (ρ = 0.82, *p* < 0.001).

DPS‐derived GFAP concentrations were substantially lower than plasma values, with a median DPS‐to‐plasma ratio of 0.18 (IQR 0.16–0.20), corresponding to an approximately fivefold reduction. Linear regression analysis demonstrated a slope of 0.16 (95% CI 0.14–0.17), indicating a consistent proportional relationship between DPS and plasma GFAP concentrations.

All samples were within the calibration curve range (4990–0.319 pg/mL; 100–0.0064 pM) and above the assay lower limit of quantification (LLoQ), including the DPS samples. The median coefficient of variation (CV) across all duplicate samples was 2.7%, indicating excellent intra‐assay precision. When mean values of duplicate measurements were used to assess the linear relationship between matrices, GFAP concentrations in plasma and DPS showed a strong positive association across diagnostic groups, including RRMS, NMOSD, MOGAD, and HC (*r* = 0.91, *p* < 0.001; Figure 2B). These findings indicate that although DPS‐derived GFAP concentrations are, due to pre‐analytical factors including sample dilution during extraction, systematically lower than plasma values, relative differences and rank‐order consistency between matrices are preserved.

### 3.4 Cross‐Platform Correlation of GFAP Quantification in AQP4‐IgG ^+^ NMOSD

To assess cross‐platform comparability of GFAP measurements in NMOSD, paired serum, plasma, and DPS samples were analyzed using Simoa (Quanterix) and NULISA platform (*n* = 10). GFAP concentrations measured in serum by Simoa showed strong correlations with plasma GFAP quantified by NULISA (*r* = 0.98, *p* < 0.001) and with DPS‐derived GFAP measured by NULISA (*r* = 0.97, *p* < 0.001). Plasma and DPS GFAP concentrations quantified by NULISA were likewise strongly correlated (*r* = 0.98, *p* < 0.001). Together, these results demonstrate high cross‐platform and cross‐matrix concordance of GFAP measurements in AQP4‐IgG^+^ NMOSD (Figure 2C).

### 3.5 GFAP Is Elevated in NMOSD Across Plasma and DPS

**FIGURE 3:**
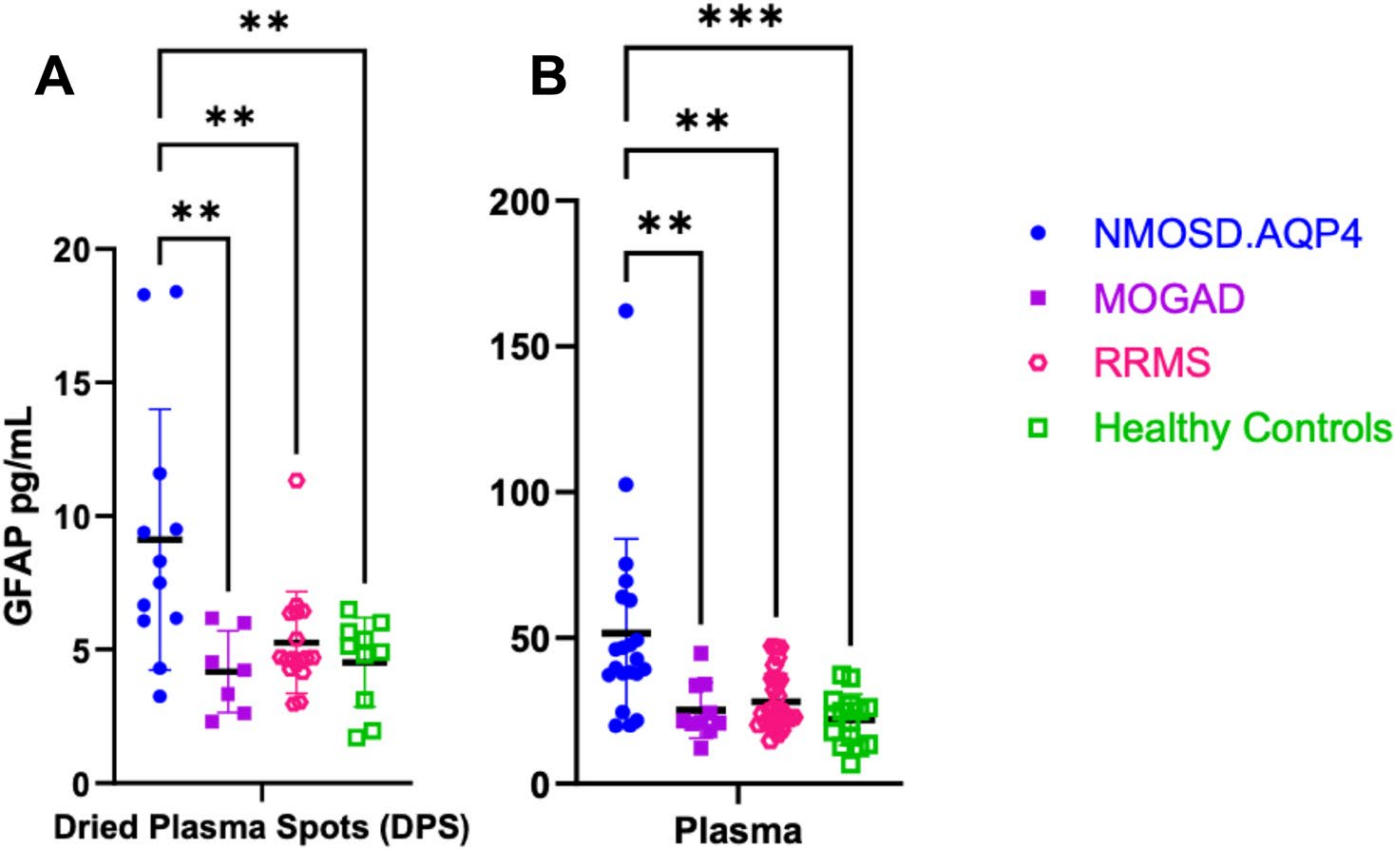
DPS and plasma GFAP concentrations across diagnostic groups. GFAP levels measured in (A) DPS and (B) plasma using the NULISA assay from individuals with NMOSD, MOGAD, RRMS, and HC. Each symbol represents an individual participant; plotted are individual values, black lines show the median and whiskers denote the interquartile range (IQR). Group comparisons were performed using the Kruskal–Wallis test (DPS: NMOSD *n* = 12, MOGAD *n* = 7, RRMS *n* = 17, HC *n* = 10; H = 21.53, *p* < 0.001; plasma: NMOSD *n* = 19, MOGAD *n* = 9, RRMS *n* = 28, HC *n* = 14; H = 21.87, *p* < 0.001), followed by Dunn's post hoc test with Bonferroni correction. GFAP concentrations were significantly higher in NMOSD compared with MOGAD (*p* = 0.0027), RRMS (*p* = 0.0032), and HC (*p* = 0.0019) for DPS, with similar elevations observed in plasma (*p* = 0.0023, *p* = 0.0030, and *p* < 0.001, respectively). No other intergroup differences were detected (all *p* > 0.9). **p < 0.01, ***p < 0.001.

To test differences in GFAP levels across diagnostic groups, group comparisons were performed using the Kruskal–Wallis test followed by Dunn's multiple‐comparisons test. GFAP concentrations measured in DPS and plasma differed significantly across the four diagnostic groups (NMOSD, MOGAD, RRMS, and HC; DPS: Kruskal–Wallis H = 21.53, *p* < 0.001; plasma: H = 21.87, *p* < 0.001). In DPS samples, GFAP concentrations were higher in the NMOSD group compared with MOGAD (*p* = 0.0027), RRMS (*p* = 0.0032), and HC (*p* = 0.0019) (Figure 3A). Similarly, plasma GFAP concentrations were elevated in NMOSD compared with MOGAD (*p* = 0.002), RRMS (p = 0.003), and HC (*p* < 0.001) (Figure 3B). No differences were observed among the remaining diagnostic groups (all *p* > 0.9).

**FIGURE 4:**
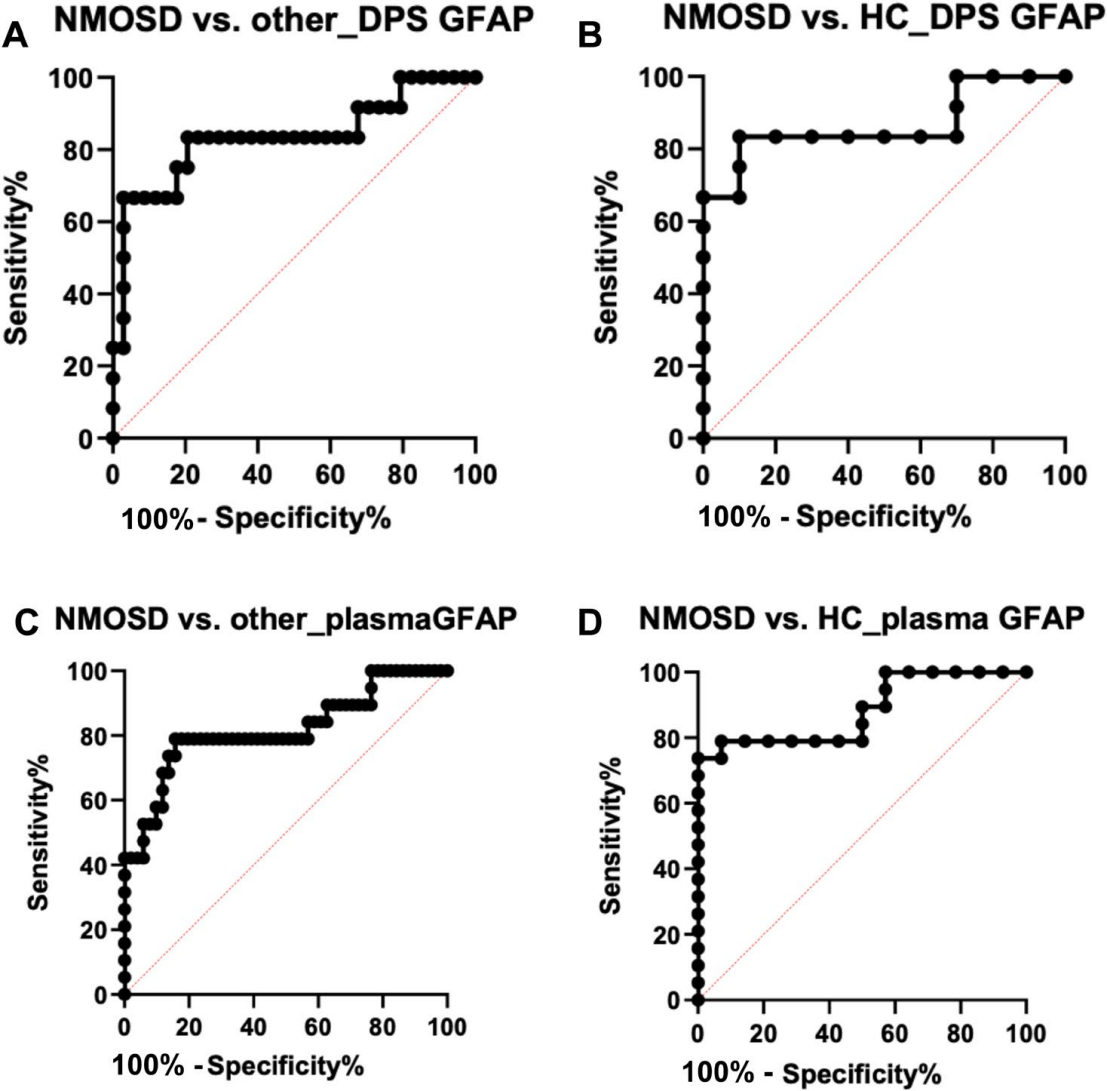
Diagnostic performance of GFAP measured in plasma and DPS for NMOSD. (A) ROC curve for DPS GFAP distinguishing NMOSD (*n* = 12) from other neurological controls (*n* = 34), showing good accuracy (AUC = 0.8333; 95% CI 0.6751–0.9915; *p* < 0.001). (B) ROC curve for DPS GFAP distinguishing NMOSD (*n* = 12) from healthy controls (*n* = 10). DPS GFAP showed good discriminatory accuracy (AUC = 0.8667; 95% CI 0.7051–1.000; *p* = 0.0037). (C) ROC curve for plasma GFAP distinguishing NMOSD (*n* = 12) from other neurological controls (*n* = 34), demonstrating good discriminatory accuracy (AUC = 0.8173; 95% CI 0.6899–0.9447; *p* < 0.0001). (D) ROC curve for plasma GFAP differentiating NMOSD (*n* = 12) from healthy controls (*n* = 10), demonstrating very good discriminatory performance (AUC = 0.8835; 95% CI 0.7682–0.9987; *p* < 0.0002).

To evaluate the diagnostic performance of GFAP for NMOSD, ROC curve analyses were performed. DPS‐derived GFAP concentrations discriminated NMOSD from other neurological controls with good accuracy (AUC = 0.8333, 95% CI 0.6751–0.9915, *p* < 0.001; Figure 4A). For this comparison, a DPS GFAP cut‐off of 6.04 pg/mL yielded a sensitivity of 83.3% (95% CI 55.2%–97.0%) and specificity of 79.4% (95% CI 63.2%–89.7%), while a higher threshold of 6.66 pg/mL increased specificity to 97.1% (95% CI 85.1%–99.9%) at a sensitivity of 66.7%. A similar discriminatory performance was observed in a restricted comparison between NMOSD and HC demonstrating good diagnostic accuracy (AUC = 0.8667, 95% CI 0.7051–1.000, *p* = 0.0037; Figure 4B). In this setting, a DPS GFAP cut‐off of 6.04 pg/mL yielded a sensitivity of 83.3% (95% CI 55.2%–97.0%) and specificity of 90.0% (95% CI 59.6%–99.5%), whereas a higher threshold of 6.58 pg/mL achieved complete specificity (100%) at a sensitivity of 66.7%.

Comparable diagnostic performance was observed for plasma‐derived GFAP concentrations. Plasma GFAP discriminated NMOSD from other neurological controls with good accuracy (AUC = 0.8173, 95% CI 0.6899–0.9447, *p* < 0.0001; Figure 4C) and differentiated NMOSD from healthy controls with very good diagnostic performance (AUC = 0.8835, 95% CI 0.7682–0.9987, *p* < 0.0002; Figure 4D).

### 3.6 Association of DPS GFAP With Clinical Disease Severity in NMOSD

**FIGURE 5:**
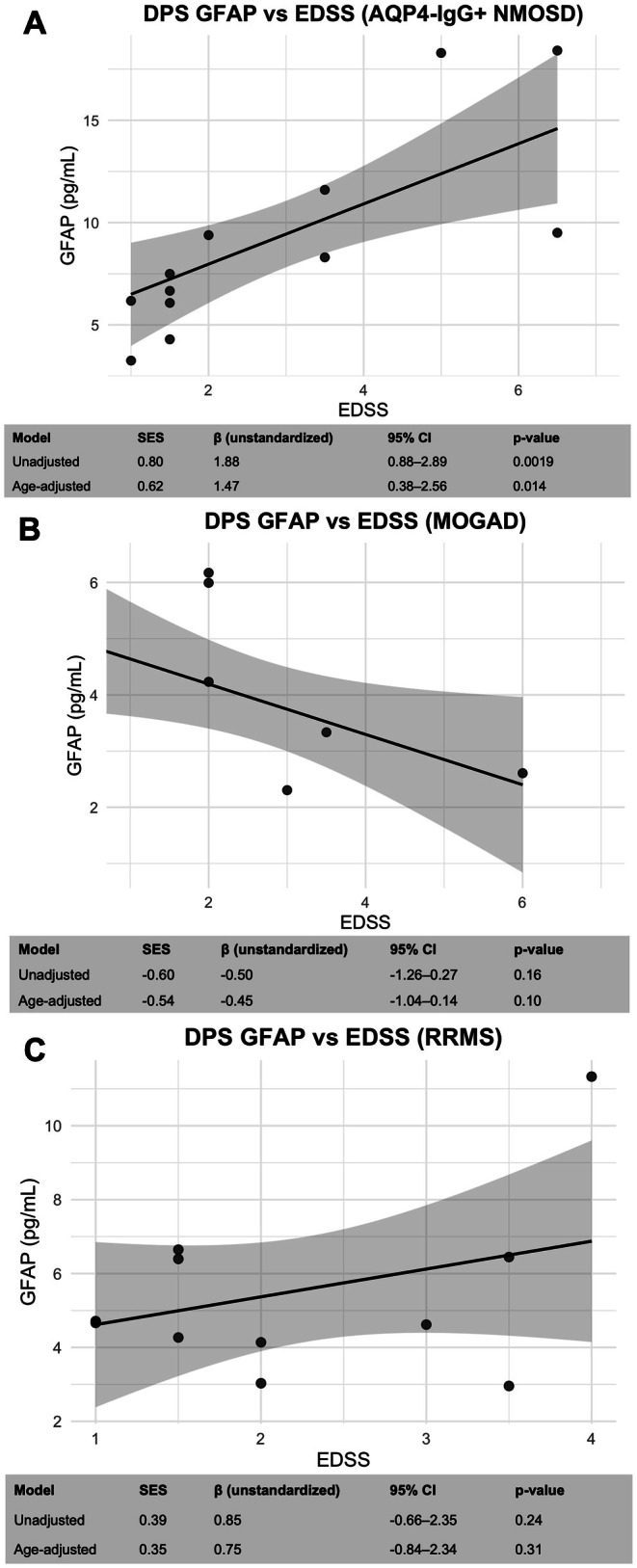
Association between DPS GFAP concentrations and disability across diagnostic groups. Scatter plots show the relationship between DPS GFAP concentrations and EDSS in (A) AQP4‐IgG^+^ NMOSD, (B) MOGAD, and (C) RRMS. Solid lines represent linear regression fits with shaded areas indicating 95% confidence intervals. In AQP4‐IgG^+^ NMOSD, DPS GFAP was positively associated with EDSS in both unadjusted (β = 1.88 pg/mL per EDSS point, 95% CI 0.88–2.89; *p* = 0.0019; SES = 0.80) and age‐adjusted models (β = 1.47, 95% CI 0.38–2.56; *p* = 0.0138; SES = 0.62). No statistically significant associations were observed in MOGAD or RRMS in either unadjusted or age‐adjusted analyses.

To investigate whether DPS GFAP levels reflect the level of disability in AQP4‐IgG^+^ NMOSD, we analyzed the association between DPS‐derived GFAP concentrations and EDSS scores. Generalized additive models did not indicate substantial nonlinearity of the association. In univariable linear regression, DPS GFAP levels were associated with EDSS (β = 1.88 pg/mL per EDSS point, 95% CI 0.88–2.89, *p* = 0.0019), corresponding to a large standardized effect size (SES = 0.80; Figure 5A). A similar association was observed for plasma GFAP. Plasma GFAP concentrations were positively associated with EDSS (β = 9.01 pg/mL per EDSS point, 95% CI 1.2–16.81; *p* = 0.0263), with a moderate‐to‐large standardized effect size (SES = 0.51).

To account for potential confounding by age, multivariable linear regression models including age as a covariate were performed. After adjustment for age, the association between DPS GFAP and EDSS remained statistically significant, although modestly attenuated (adjusted β = 1.47 pg/mL per EDSS point, 95% CI 0.38–2.56, *p* = 0.014; SES = 0.62).

In contrast, no significant associations between DPS GFAP levels and EDSS were observed in MOGAD (unadjusted β = −0.50, 95% CI −1.26 to 0.27, *p* = 0.16; age‐adjusted β = −0.45, 95% CI −1.04 to 0.14, *p* = 0.10) or RRMS (unadjusted β = 0.85, 95% CI −0.66 to 2.35, *p* = 0.24; age‐adjusted β = 0.75, 95% CI −0.84 to 2.34, *p* = 0.31), with small to moderate effect sizes (Figure 5B,C).

## 4 Discussion

In this study, we investigated whether GFAP can be quantified from DPS samples and retain clinical relevance in NMOSD. Using an ultra‐sensitive proximity ligation‐based immunoassay, we assessed analytical feasibility, concordance with conventional venous blood matrices, disease specificity, and associations with neurological disability. Reliable biomarker quantification from DPS represents an essential analytical prerequisite for future minimally invasive sampling approaches, including capillary or self‐sampling devices. Establishing such approaches for biomarker assessment is clinically relevant in NMOSD, a rare but severe autoantibody‐mediated astrocytopathy in which access to specialized care, repeated venipuncture, autoantibody diagnostics and longitudinal biomarker monitoring remain challenging, particularly outside specialized referral centers [3, 16].

GFAP is a well‐established marker of astrocytic injury and has emerged as a key blood‐based biomarker in AQP4‐IgG^+^ NMOSD. Multiple independent studies have consistently demonstrated elevated circulating blood GFAP levels in NMOSD compared with other neuroinflammatory diseases, as well as associations with disease activity and neurological disability [4, 5, 6, 7, 23]. In agreement with this body of evidence, we observed significantly higher GFAP concentrations in DPS and plasma in NMOSD than in MOGAD, RRMS, and HC, whereas no relevant differences were detected among non‐NMOSD disease groups. These findings further support the specificity of GFAP as a biomarker reflecting astrocytic pathology rather than generalized neuroinflammation, consistent with the distinct pathophysiological mechanisms underlying NMOSD compared with other demyelinating disorders [24].

A central contribution of this study is the demonstration that DPS sampling preserves biologically meaningful GFAP information in NMOSD, MOGAD, and MS. Although absolute GFAP concentrations were systematically lower in DPS than in plasma due to preanalytical factors related to extraction efficiency and dilution, this difference followed a consistent proportional pattern, with a median DPS‐to‐plasma ratio of approximately 0.18, corresponding to an approximately fivefold reduction. Linear regression analysis further supported this relationship, demonstrating a slope of 0.16 between DPS and plasma GFAP concentrations. Despite these systematic differences in absolute levels, strong correlations and preserved rank‐order relationships were observed across matrices. Importantly, the preservation of NMOSD‐associated GFAP elevations across both DPS‐ and plasma‐derived GFAP measurements quantified by NULISA suggests that DPS‐based measurements retain biologically meaningful diagnostic group differences despite lower absolute analyte concentrations. Notably, the same pattern of significantly elevated GFAP concentrations in NMOSD compared with MOGAD, RRMS, and healthy controls was consistently observed across both matrices. These findings support the concept that DPS‐derived GFAP primarily reflects preserved relative disease‐associated differences and rank‐order relationships rather than absolute concentrations directly interchangeable with conventional plasma or serum measurements. In line with this, ROC analyses demonstrated that DPS‐derived GFAP retained good discriminatory performance for distinguishing AQP4‐IgG^+^ NMOSD from other diagnostic groups comparable to plasma‐derived GFAP. Similar observations were recently reported for GFAP and other protein biomarkers in dried blood or plasma spot matrices obtained by minimal‐invasive capillary sampling in Alzheimer's disease research using Simoa [8, 9]. In addition, we observed strong cross‐platform concordance between blood GFAP measurements obtained using the NULISA and Simoa technology. This supports the analytical robustness of proximity ligation‐based GFAP quantification in DPS. The ultra‐sensitive analytical performance of the NULISA platform, enabling protein quantification in the attomolar range, is likely of particular importance for DPS‐based biomarker assessment, where dilution and matrix‐related extraction effects may substantially reduce analyte concentrations.

DPS‐derived GFAP levels were, similar to plasma GFAP, strongly associated with neurological disability as measured by EDSS in NMOSD, with large standardized effect sizes and a linear relationship. The association remained significant after adjustment for age, indicating that the observed relationship was not solely driven by age, a known determinant of circulating GFAP levels [25, 26]. This suggests that DPS GFAP reflects disease‐specific burden and cumulative astrocytic injury, in line with previous reports linking circulating serum GFAP to disability and disease severity in NMOSD [5, 7, 23].

A reliable DPS‐based GFAP assessment may have particular relevance for disease monitoring in NMOSD. GFAP elevations have been shown to precede or accompany clinical relapses, including evidence from the N‐MOmentum trial [4]. In addition, growing evidence suggests that NMOSD may exhibit ongoing tissue damage and subclinical disease activity outside clinically manifest relapses, including progressive retinal neuroaxonal loss and structural CNS changes associated with blood GFAP [27, 28, 29]. While the present study was not designed to assess subclinical activity, the current findings establish the analytical feasibility and clinical relevance of DPS‐derived GFAP measurements, providing a foundation for future longitudinal investigations using minimally invasive capillary sampling. Such sampling approaches have the potential to reduce resource‐intensive steps associated with conventional venipuncture. Compared with conventional plasma analytics, DPS offers simplified sample handling and capillary‐based collection, which may improve accessibility for frequent or short‐interval monitoring for patients with impaired mobility, and in settings where repeated venipuncture, cold‐chain logistics, and outpatient visits are difficult to realize. From a broader perspective, these features are globally particularly relevant for NMOSD, where delayed diagnosis and limited availability to biomarker‐based diagnostics remain major challenges in many regions, including parts of the Global South [11, 12]. This challenge is particularly evident in parts of Africa, where a recent review summarizing more than 600 published cases suggests that NMOSD remains substantially under‐recognized, likely owing to limited access to neurological care and biomarker testing [13]. The need for accessible and low‐cost diagnostic strategies is therefore especially pronounced in such settings. Scalable approaches such as capillary DPS may help narrow such diagnostic gaps, consistent with recent work showing that dried blood spot‐based methods can improve global access to AQP4‐IgG testing [30]. The present study, however, was performed under controlled laboratory conditions using biobanked frozen and thawed plasma and DPS samples, both derived by conventional venipuncture, and does not directly demonstrate feasibility in decentralized, home‐based settings. Future studies evaluating capillary sampling workflows under prospective real‐world conditions will be necessary to determine the practical clinical utility and scalability of minimally invasive DPS‐based GFAP assessment in such settings.

Several limitations should be considered when interpreting these findings. These include the relatively small sample size and age differences between disease groups. The NMOSD group was older than the other groups. As age influences circulating GFAP levels, residual confounding cannot be excluded. However, the association between plasma GFAP and EDSS remained statistically significant after adjustment for age. A limitation relates to pre‐analytical variability in DPS processing, with a substantial proportion of samples excluded due to extraction‐related effects, including plate edge variability and incomplete recovery. This indicates current limitations in workflow robustness. Potential mitigation strategies include optimization of elution and extraction protocols, avoidance of edge wells during plate processing, and use of less absorbent collection materials, which were not systematically evaluated in this study. The relatively small DPS subgroup limits the robustness and generalizability of the diagnostic performance analyses, and the incremental value over established plasma‐ or serum‐based GFAP measurements should therefore be interpreted cautiously. Additionally, all NMOSD patients were clinically stable and receiving B‐cell–depleting therapy, a context in which even lower GFAP levels would be expected [31]. DPS sampling was performed under controlled conditions, and minimally invasive capillary sampling was not evaluated. To address this limitation in future studies, minimally invasive capillary sampling should be used as for example, recently validated for GFAP quantification in the context of Alzheimer's disease [9]. Furthermore, the cross‐sectional design precludes conclusions regarding temporal dynamics, including assessments of subclinical disease activity in NMOSD. Larger prospective, longitudinal, and multicenter studies will be required to further validate this approach. Specifically, studies incorporating capillary sampling in relapse‐associated and relapse‐free intervals together with complementary MRI‐ and optical coherence tomography‐based measures of subclinical tissue injury will be important to further define the temporal dynamics and clinical utility of DPS‐derived GFAP assessment in NMOSD.

In conclusion, our findings demonstrate that GFAP can be quantified from DPS samples using NULISA AQ and preserves diagnostic and clinical relevance in NMOSD. DPS‐based GFAP assessment represents a promising strategy for biomarker evaluation in NMOSD, with potential future applications using minimally invasive capillary blood sampling for diagnosis and disease monitoring. While further methodological refinement and longitudinal evaluation—including validation using capillary‐derived samples—are required before clinical implementation, this approach may contribute to more accessible and frequent biomarker assessment in NMOSD and related astrocytopathic disorders.

## Author Contributions

**Felix Wohlrab:** conceptualization, data curation, formal analysis, investigation, methodology, project administration, writing – original draft. **Evelyn Alvarez:** investigation, writing – review and editing. **Roua Hamdi:** data curation, formal analysis, writing – review and editing. **Bingqing Zhang:** investigation, writing – review and editing. **Hayeun Ji:** investigation, writing – review and editing. **Niyati Jhaveri:** investigation, writing – review and editing. **Patrick Schindler:** validation, writing – review and editing. **Pedro Sanchez:** investigation. **Pia S. Sperber:** investigation. **Tanja Schmitz‐Hübsch:** investigation. **Frederike C. Oertel:** investigation. **Nisa Vorasoot:** writing – review and editing. **Najib Kissani:** writing – review and editing. **Falko Böhringer:** conceptualization, resources, writing – review and editing. **Jens Kuhle:** validation, writing – review and editing. **Anne‐Katrin Pröbstel:** validation, writing – review and editing. **Xiao‐Jun Ma:** investigation, writing – review and editing. **Péter Körtvelyessy:** conceptualization, resources, writing – review and editing. **Patrick Waters:** formal analysis, writing – review and editing. **Friedemann Paul:** conceptualization, resources, supervision, writing – original draft.

## Funding

The authors have nothing to report.

## Conflicts of Interest

Felix Wohlrab: Current research grants by Bundesministerium für Bildung und Forschung (BMBF), Carstens Stiftung and Alexion Pharma. Received travel funding and/or speaker honoraria from UCB and Sobi. Roua Hamdi: The author declares no conflicts of interest. Evelyn Alvarez, Bingqing Zhang, Hayeun Ji, Niyati Jhaveri, and Xiao‐jun Ma are employees and shareholders of Alamar Biosciences. Pedro Sanchez: The author declares no conflicts of interest. Tanja Schmitz‐Hübsch: The author declares no conflicts of interest. Nisa Vorasoot: The author declares no conflicts of interest. Najib Kissani: The author declares no conflicts of interest. Jens Kuhle: Jens Kuhle received speaker fees, research support, travel support, and/or served on advisory boards by Swiss MS Society, Swiss National Research Foundation (320030_212534/1), University of Basel, Progressive MS Alliance, Alnylam, Argenx, Bayer, Biogen, Bristol Myers Squibb, Celgene, Immunic, Merck, Neurogenesis, Novartis, Octave Bioscience, Quanterix, Roche, Sanofi, Stata DX. Péter Körtvelyessy and Falko Böhringer: are employed by Labor Berlin—Charité Vivantes Services GmbH. This employment constitutes a potential conflict of interest as it may be perceived as influencing the author's objectivity. The authors declare that there are no additional financial or nonfinancial relationships that could be construed as a potential conflict of interest, including patent or stock ownership, membership on a company board of directors, membership on advisory boards or committees, consultancy activities, or receipt of speaker's fees. Frederike C. Oertel: Current research grants by the German Research Foundation (DFG, TRR418), Hertie foundation, German ME/CFS foundation, Bundesministerium für Bildung und Forschung (BMBF), Novartis and UCB—not related to this project. Past fellowship support by the American Academy of Neurology and the National MS Society (until 2023). Past research grant by the DGN (Germany Neurology Association) and DFG‐TWAS program. Speaker honoraria by UCB and Novartis. Travel support by Guthy Jackson Charitable Foundation, European Committee for Research and Treatment in Multiple Sclerosis and American Academy of Neurology. Academic editor at Neurology, DGNeurologie and Neurological Research & Practice. Board member at the IMSVISUAL consortium. Friedemann Paul: served on the scientific advisory boards of Novartis and MedImmune; received travel funding and/or speaker honoraria from Bayer, Novartis, Biogen, Teva, Sanofi‐Aventis/Genzyme, Merck Serono, Alexion, Chugai, MedImmune, and Shire; is an associate editor of Neurology: Neuroimmunology & Neuro‐inflammation; is an academic editor of PLoS ONE; con‐sulted for Sanofi Genzyme, Biogen, MedImmune, Shire, and Alexion; received research support from Bayer, Novartis, Biogen, Teva, Sanofi‐Aventis/Geynzme, Alexion, and Merck Serono; and received research support from the German Research Council, Werth Stiftung of the City of Cologne, German Ministry of Education and Research, Arthur Arnstein Stiftung Berlin, EU FP7 Framework Pro‐gram, Arthur Arnstein Foundation Berlin, Guthy‐Jackson Charitable Foundation, and NMSS. F. Paul is also supported by Deutsche Forschungsgemeinschaft (DFG Exc 257), Bundesministerium für Bildung und Forschung (Competence Network Multiple Sclerosis KKNMS) and the Guthy Jackson Charitable Foundation. Pia Sophie Sperber: The author declares no conflicts of interest. Patrick Schindler: received speaker's honoraria, travel support and/or served on advisory boards by Alexion, Roche, and UCB. Patrick Waters: is a named inventor on patents for antibody assays (WO/2010/046716) with royalties paid by Euroimmun AG and disease biomarker patents (WO2019211633A1, WO2022189788A1). He has received honoraria from Biogen Idec, Mereo Biopharma, Retrogenix, UBC, Euroimmun AG, UCB, F. Hoffmann La‐Roche, Forum for Indian Neurology Education (FINE) and Alexion; travel grants from the Guthy‐Jackson Charitable Foundation; and research funding from Euroimmun AG and the Guthy‐Jackson Charitable Foundation. His work in the Oxford Autoimmune Neurology Diagnostic Laboratory is partly supported by the NHS Commissioning service for NMOSD. He serves on the editorial boards of Neurology, Neuroimmunology & Neuroinflammation and the Journal of Clinical Neurology. Anne‐Katrin Pröbstl: Dr. Pröbstel has received financial compensation for participation in advisory boards and/or consultations from Biogen, Chugai, Novartis, Roche, and UCB. Der Pröbstel serves as the deputy editor of Neurology, Neuroimmunology, Neuroinflammation.

## Data Availability

The data supporting the findings of this study were collected within the BERLimmun registry at Charité‐Universitätsmedizin Berlin. Due to ethical and data protection restrictions associated with the approved study protocol and participant consent, individual‐level data cannot be made publicly available. De‐identified data and the statistical analysis code used in this study may be made available from the corresponding author upon reasonable request, subject to approval by the responsible ethics committee, institutional regulations, and applicable data protection laws.
